# Evaluation of brain structure and metabolism in currently depressed adults with a history of childhood trauma

**DOI:** 10.1038/s41398-022-02153-z

**Published:** 2022-09-17

**Authors:** Joshua S. Jones, Samantha J. Goldstein, Junying Wang, John Gardus, Jie Yang, Ramin V. Parsey, Christine DeLorenzo

**Affiliations:** 1 University of Rochester, Rochester, NY USA; 2 Department of Psychiatry and Behavioral Science, Stony Brook University, New York, NY USA; 3 Department of Applied Mathematics and Statistics, Stony Brook University, New York, NY USA; 4 Department of Family, Population & Preventive Medicine, Stony Brook University, New York, NY USA; 5 Department of Biomedical Engineering, Stony Brook University, New York, NY USA

**Keywords:** Neuroscience, Depression

## Abstract

Structural differences in the dorsolateral prefrontal cortex (DLPFC), anterior cingulate cortex (ACC), hippocampus, and amygdala were reported in adults who experienced childhood trauma; however, it is unknown whether metabolic differences accompany these structural differences. This multimodal imaging study examined structural and metabolic correlates of childhood trauma in adults with major depressive disorder (MDD). Participants with MDD completed the Childhood Trauma Questionnaire (CTQ, *n* = 83, *n* = 54 female (65.1%), age: 30.4 ± 14.1) and simultaneous positron emission tomography (PET)/magnetic resonance imaging (MRI). Structure (volume, *n* = 80, and cortical thickness, *n* = 81) was quantified from MRI using Freesurfer. Metabolism (metabolic rate of glucose uptake) was quantified from dynamic ^18^F-fluorodeoxyglucose (FDG)-PET images (*n* = 70) using Patlak graphical analysis. A linear mixed model was utilized to examine the association between structural/metabolic variables and continuous childhood trauma measures while controlling for confounding factors. Bonferroni correction was applied. Amygdala volumes were significantly inversely correlated with continuous CTQ scores. Specifically, volumes were lower by 7.44 mm^3^ (95% confidence interval [CI]: –12.19, –2.68) per point increase in CTQ. No significant relationship was found between thickness/metabolism and CTQ score. While longitudinal studies are required to establish causation, this study provides insight into potential consequences of, and therefore potential therapeutic targets for, childhood trauma in the prevention of MDD. This work aims to reduce heterogeneity in MDD studies by quantifying neurobiological correlates of trauma within MDD. It further provides biological targets for future interventions aimed at preventing MDD following trauma. To our knowledge, this is the first simultaneous positron emission tomography (PET) and magnetic resonance imaging (MRI) study to assess both structure and metabolism associated with childhood trauma in adults with MDD.

## Introduction

Major depressive disorder (MDD) is the leading cause of disability worldwide, however, early detection and intervention are hindered by the limited knowledge of MDD’s underlying biology [1]. The presence of childhood trauma, such as sexual abuse [2], emotional abuse [3], and family conflict [4] are significantly associated with MDD and the prevalence of childhood trauma in depressed patients is reported to be as high as 75% [5–7], though the biological mechanism linking them is unknown.

One possible mechanism may be through childhood trauma’s detrimental effects on the developing brain, including in emotional/stress regulation and network architecture [8]. Specifically, childhood trauma can lead to improper neurodevelopment of the hypothalamic pituitary adrenal (HPA) axis and corticolimbic circuits [9, 10]. Critical regions in this circuitry are the dorsolateral prefrontal cortex (DLPFC) and anterior cingulate cortex (ACC), which are responsible for coordinating responses to negative stimuli [9, 11, 12]. The hippocampus and amygdala, while not explicitly part of the HPA axis, help facilitate these responses [9, 11, 12] and are also known to play an important role in memory and emotions [13, 14]. Chronic stress, such as childhood trauma, can cause long-term effects in these regions [15]. For example, humans exposed to childhood trauma exhibit smaller hippocampus [16–18] and amygdala [15, 19, 20] volumes, and animal models have shown a correspondingly lower dendritic spine density in these regions in trauma models [15, 21]. Additionally, one study in humans noted that hippocampus and amygdala volumes were inversely correlated with severity of childhood trauma [19]. Reduced volumes in humans were also observed in the DLPFC and ACC regions in those who experienced childhood trauma [8, 17, 22–27].

Though the volumetric findings appear consistent, an important open question is whether functional differences are also associated with childhood trauma. Using ^18^F-fluorodeoxyglucose positron emission tomography (FDG-PET) to assess cerebral metabolism [28, 29], rhesus monkeys with childhood trauma (maternal separation after birth) were found to exhibit lower hippocampal metabolism compared to controls [9]. In humans, a functional magnetic resonance imaging (fMRI) study indicated HPA axis hypo-reactivity in adults who experienced childhood trauma similar to that reported in animal models [30]. Other human fMRI studies demonstrated that neuronal activity is decreased in areas such as the PFC and amygdala in adults [8, 31, 32] and children [22] who experienced childhood trauma, however, not all study results have been consistent. For example, some adult fMRI studies report amygdala hyperreactivity in those who experienced childhood trauma [15, 20, 21, 33, 34]. To our knowledge, no PET studies examining brain metabolism in adults who have experienced childhood trauma have been reported, and of the fMRI studies, many have less than 30 participants [8, 22, 30, 31, 33] per cohort.

Regions of the brain implicated in childhood trauma, e.g., the DLPFC, ACC, hippocampus, and amygdala, have also been noted in MDD. However, structural results are not consistent, with studies reporting lower [24, 35, 36], greater [37–39], or, as we and others previously reported, no significant differences [40–43] in thickness or volume of these regions between depressed individuals and non-depressed controls. In terms of function, Baeken et al. reported increased metabolic activity in the dorsomedial PFC in those with MDD compared to controls [44] but a meta-analysis of neuroimaging studies using FDG-PET found that MDD patients had significantly lower regional cerebral glucose metabolism in the ACC, hippocampus and other regions when compared with healthy controls [45, 46].

The equivocal MDD studies above may have been confounded by the prevalence of childhood trauma within MDD. However, the neurobiology of childhood trauma within MDD remains particularly elusive and is therefore the topic of this study. Two studies found smaller hippocampus and orbitofrontal cortical volumes in childhood trauma independent of MDD diagnosis [47, 48]. However, the volumes of these regions were not found to be associated with childhood trauma in the mega-analysis of 3036 subjects (958 of which had MDD) by the ENIGMA-MDD network. In that study, a significant inverse association between the severity of childhood trauma and volumes was only found in the caudate nucleus [49]. As cortical thickness reflects different neuroanatomic properties than volume [50], a separate study examined cortical thickness in this population, implicating regions such as the PFC and ACC [51]. This study found that childhood trauma severity was associated with increased cortical thickness in the rostral ACC and decreased cortical thickness in the temporal and parietal lobes compared to controls. However, to our knowledge, no study to date has examined the metabolic activity of these regions in MDD with childhood trauma.

To address these open questions, we propose an analysis of the metabolism of the amygdala, ACC, hippocampus, and DLPFC through FDG-PET in addition to a structural MRI in the same participants with MDD and varying levels of childhood trauma. We hypothesize that volume and cortical thickness as quantified by MRI and metabolism as quantified by FDG-PET in these regions will be inversely related to childhood trauma severity. As thickness and volume can be affected independently by childhood trauma, these variables are analyzed separately and hypothesized to have different relationships with childhood trauma. This study would, for the first time, assess both structure, using MRI, and function, using FDG-PET, acquired simultaneously, of critical regions relating to the stress response in MDD with childhood trauma.

## Methods and materials

### Participants

This study was approved by the Institutional Review Board of Stony Brook University. All participants provided informed consent and were recruited as a community sample as described in Hill et al. [52]. Data from this study (Advancing Personalized Antidepressant Treatment Using PET/MRI, ClinicalTrials.gov, NCT02623205) has been previously published; however, none of the previous studies examined childhood trauma; they involved PET-only measures [52], or only magnetic resonance spectroscopy [53]. Inclusion criteria include ability to provide informed consent, age of at least 18 years old, diagnosis of current major depressive episode (MDE; confirmed by SCID-IV interview), and a score of at least 22 on the Montgomery-Asberg Depression Rating Scale (MADRS; 22 is considered moderate depression) [54]. Participants were excluded under the following conditions: currently efficaciously treated with antidepressants, contraindications to escitalopram including previous failure of escitalopram therapy (the participants from this study were later treated with escitalopram [52]), electroconvulsive therapy (ECT) within 6 months, lifetime history of psychosis or bipolar disorder, actively suicidal, high potential for excessive substance use during the study period (decided by the clinician in conjunction with the study team on an individual basis with reference to the participant’s clinical interview, lifestyle, frequency of current substance use, protective factors and other related information), significant active physical illness, significant neurological deficits, or contraindications to MRI or PET imaging including metal implants or pregnancy.

### Clinical measures

Participants were first screened over the phone by a study team member to determine interest in the study and eligibility. Participants were then assessed by a clinician (psychiatric nurse practitioner or psychiatrist) and a trained rater (psychologist or trained staff). Participants were either psychotropic medication naive (*n* = 44; 53.0%) or psychotropic medication free for at least 3 weeks before imaging (*n* = 39; 47.0%). 69.9% of participants (*n* = 58) underwent treatment with psychotherapy while 30.1% (*n* = 25) did not. Participants who had previously been on psychotherapy were allowed to continue.

Washout (if needed) was completed over a maximum of 4 weeks before the 3-week psychotropic medication-free period. Following the medication-free period, participants still meeting eligibility criteria were scheduled for simultaneous PET/MRI imaging with ^18^F-fluorodeoxyglucose (FDG) on a Siemens Biograph mMR (Siemens, Erlangen, Germany). All MRI and PET analyses were performed by technicians blinded to participant condition.

### Childhood trauma

All participants completed the Childhood Trauma Questionnaire (CTQ) [55]. Scores for each dimension range from 5 to 25; higher scores indicate more severe maltreatment [6, 47, 55, 56]. Based on Table 1, a new, discrete childhood trauma level was defined as follows: none (0): participants with “none” (0) across all trauma categories; low (1): participants who have at least one “low” value in a trauma category and no “moderate” or “severe” scores; moderate (2): participants who have at least one “moderate” value in a trauma category and no severe scores; severe (3): participants who have at least one ‘severe’ value in a trauma category.

**Table 1:** Childhood Trauma Questionnaire by discrete categories of childhood trauma as defined by Bernstein et al. [55]. These definitions were used to calculate the discrete childhood trauma levels.

CTQ subscale	None	Low	Moderate	Severe
Emotional abuse	0–8	9–12	13–15	16+
Physical abuse	0–7	8–9	10–12	13+
Sexual abuse	0–5	6–7	8–12	13+
Emotional neglect	0–9	10–14	15–17	18+
Physical neglect	0–7	8–9	10–12	13+

### Demographics

85 participants qualified for and were interested in the study (*n* = 29 males and *n* = 56 females, see CONSORT diagram in Hill et al. [52]) and were scheduled for imaging. Two participants were excluded from analysis since they were missing physical neglect (*n* = 1), sexual abuse and emotional neglect (*n* = 1) subscale items in the CTQ. Additionally, PET imaging for 13 participants was excluded (>20% change in blood glucose over scan duration, *n* = 11, diabetes, *n* = 1, or uncorrectable motion, *n* = 1). For one participant, excessive motion was noted in the MRI preventing it from being processed, or the left hippocampus segmentation failed (other regions acceptable, *n* = 1) or the cortical region segmentation failed due to exclusion of the temporal pole (subcortical regions acceptable, *n* = 1). MRI exclusions were based on visual inspection of the MRI and outliers from the Freesurfer analysis, as a slice-by slice inspection of Freesurfer parcellation results in negligible effects on outcome [57]. The demographic/clinical characteristics of the participants are reported in Table 2. Comorbidities included Posttraumatic Stress Disorder (*n* = 13; 15.85%), Obsessive Compulsive Disorder (*n* = 1; 1.22%), other anxiety disorder (*n* = 61; 76.25%) or dysthymia (*n* = 46; 56.10%).

**Table 2:** Means and standard deviations (SD) of demographic and clinical characteristics by discrete categories of childhood trauma (childhood trauma) as defined in Table 1.

*N* = 83	CTQ total (*n* = 83) Mean (SD)	CTQ none (*n* = 13) Mean (SD)	CTQ low (*n* = 11) Mean (SD)	CTQ moderate (*n* = 25) Mean (SD)	CTQ severe (*n* = 34) Mean (SD)
Male					
(*n* = 29)	46.2 (14.1)	27.3 (1.7)	33.6 (4.0)	44.0 (2.5)	60.5 (9.3)
Female					
(*n* = 54)	48.5 (17.6)	27.2 (1.6)	35.0 (5.4)	44.6 (4.5)	63.0 (16.0)
Age	30.4 (14.0)	31.3 (13.8)	36.0 (19.1)	27.3 (10.2)	30.6 (14.7)
HDRS-17	18.0 (4.5)	17.2 (4.5)	17.5 (5.5)	17.6 (4.7)	18.8 (4.1)
CTQ	47.7 (16.4)	27.2 (1.5)	34.4 (4.6)	44.4 (3.9)	62.2 (14.1)

^*HDRS-17* 17-Item Hamilton Depression Rating Scale.^

### Magnetic resonance imaging (MRI)

A magnetization-prepared rapid gradient-echo (MP-RAGE) T1-weighted structural image was acquired with the following parameters: TR = 2300 ms, TE = 3.24 ms, flip angle = 9 degrees, IPAT GRAPPA factor 2, FOV = 223 x 210 x 195mm, bandwidth = 220 Hz/Px, echo spacing=7.8 ms, voxel size=0.87 × 0.87 × 0.87 mm, and acquisition time = 5:40 min.

All T1 images were run through an array of quality assurance examinations for common artifacts, including slice‐wise intensity, venetian blind, ghost, gradient‐wise, and ring motion artifacts. T1 structural images were processed through Freesurfer 5.3.0 (http://surfer.nmr.mgh.harvard.edu) to automatically extract the thickness of cortical regions (bilateral ACC and DLPFC), and as well as regional volumes (bilateral hippocampus/amygdala/ACC/DLPFC) from the Desikan-Killiany atlas [58].

### Positron emission tomography (PET)

Up to 185 MBq of FDG were injected intravenously and emission data was acquired for 60 min on a Siemens Biograph mMR. Raw listmode PET data were reconstructed offline using Siemens’ e7 Tools software and a childhood trauma-like Boson MR-based attenuation map [59]. Sinogram files were generated using the following frame definitions: 8 x 15s, 6 x 30s, 5 x 60s, 4 x 300s, and 3 x 600s. Sinogram data were backprojected with filtering onto a 344×344 matrix with scatter correction and no smoothing. Frames were corrected for motion and co-registered to MRI for regional delineation. Regional time activity curves were defined and fit using a single venous blood sample and the Patlak approach with Simultaneous Estimation as described in Hill et al. [52].

### Statistical analysis

#### Models

Linear mixed models were utilized to examine the relationship between continuous levels of childhood trauma and each outcome variable (cortical thickness in cortical regions: bilateral ACC/DLPFC, volume in cortical and subcortical regions: bilateral hippocampus/amygdala/ACC/DLPFC, metabolism in cortical and subcortical regions: bilateral hippocampus/amygdala/ACC/DLPFC), after controlling for age, age^2^ (to account for non-linear effects) and sex [60], similar to the analysis performed by Bartlett et al. [61]. In Supplementary Analysis, linear mixed models were used to examine the differences between outcome variables within discrete levels of childhood trauma.

A cube root transformation was used for volume to meet the normality assumption in linear mixed models. An Unstructured variance-covariance structure for the repeated measurements was selected based on Akaike Information Criteria (AIC), and other variance-covariance structures considered included Compound Symmetric, Autoregressive (1) and Toeplitz. Bonferroni correction for multiple comparisons was performed by comparing p-values to significance level (0.05) divided by 10 (since outcomes examined included cortical thickness in two regions, volume in four regions, and metabolism in four regions).

In the continuous analysis, multiple linear regression models were further used to determine the estimated coefficient and 95% confidence interval for outcomes within the regions where significant relationships were found in linear mixed models. A Wilcoxon rank sum test was used to determine the relationship between total CTQ score and sex. For continuous variables (age, age^2^), Spearman rank correlation coefficient was used to measure the linear relationships with CTQ score and *p*-values were from t distributions with (*n* – 2) degrees of freedom. These tests were used to find the relationship between CTQ score and other exploratory variables, not for building the regression models.

As depression severity may be associated with neurobiology and to exclude this potential confounding factor, the discrete and continuous linear mixed models were repeated with and without depression severity (Table 2) as a covariate.

#### Structure/function

To examine the relationship between structure and function, a linear regression was examined between metabolic rate of glucose uptake and either thickness or volume of each region.

Statistical analysis was performed using SAS 9.4 (SAS Institute Inc., Cary, NC). Residuals were created in IBM SPSS Statistics for Macintosh, Version 26.0. (IBM Corp., Armonk, NY) and plotted in GraphPad Prism for Macintosh, Version 9.1.2 (GraphPad Software., San Diego, CA).

## Results

### Analysis using childhood trauma severity as a continuous variable

**Fig. 1:**
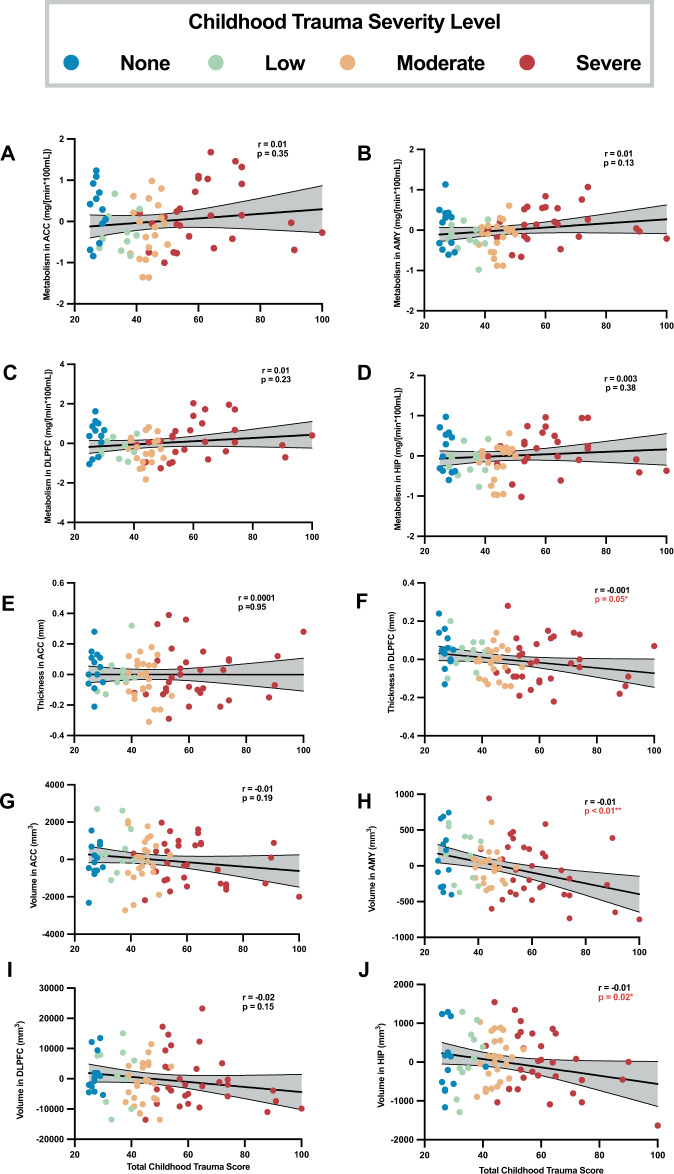
Scatterplots of the residual values calculated from regional metabolism, thickness, and volume versus total Childhood Trauma Questionnaire (CTQ) score. Residual values from multiple regression analysis of brain volume/thickness/metabolism covarying for age, age^2^, and sex are displayed on the vertical axis. The colors represent the discrete categories of childhood trauma (none, low, moderate, severe) as defined in Table 1. The regression line represents the line of best fit. The lines surrounding the gray shaded area represent the 95% confidence interval. ACC Anterior Cingulate Cortex (**A**, **E**, **G**), AMY Amygdala (**B**, **H**), DLPFC Dorsolateral Prefrontal Cortex (**C**, **F**, **I**), HIP Hippocampus (**D**, **J**).

Neurobiological variables were examined relative to the total CTQ score as a continuous measure (Fig. 1). Of the variables shown in Fig. 1, three showed significant relationships with CTQ total score, amygdala volume (Fig. 1H), hippocampus volume (Fig. 1J) and DLPFC thickness (Fig. 1F). The amygdala volume finding (*p* = 0.0009) remained significant following Bonferroni correction. For each unit increase in CTQ, amygdala volume is estimated to be lower by 7.44 mm^3^ (95% confidence interval [CI]: –12.19 to –2.68 mm^3^). For the hippocampus volume and DLPFC thickness, these values were 82.71 mm^3^ (CI:–193.66 to 28.24 mm^3^) and 0.0015 mm (CI: 0.0029 to –0.0001 mm), respectively. Differences in CTQ total scores between males and females were not significant (Wilcoxon rank sum test *p* = 0.72) and there was no significant linear relationship between CTQ and age (Spearman Correlation Coefficient = –0.09, *p* = 0.43).

No significant association was found between depression severity and CTQ score and including depression severity as a covariate did not change model results.

### Structure versus function

**Fig. 2:**
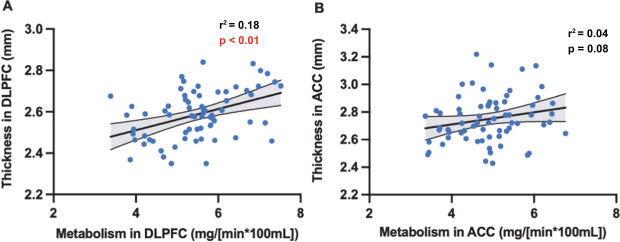
Scatterplots of regional thickness in the cortical regions versus metabolism. The regression line represents the line of best fit. The lines surrounding the gray shaded area represent the 95% confidence interval. DLPFC dorsolateral prefrontal cortex (**A**), ACC anterior cingulate cortex (**B**).

As the goal of this study was to determine the relationship of childhood trauma to brain structure and function, a natural resulting question is whether a relationship between structure and function exists within each region. Thickness and metabolism were correlated in the DLPFC (Fig. 2A, *p* < 0.001). Thickness and/or volume in the other regions were not significantly correlated with metabolism in those regions.

## Discussion

This study critically considered the correlates of childhood trauma within MDD in brain thickness, volume, and metabolism. The percentage of those with MDD who have experienced childhood trauma has been reported to be as high as 75% [5–7]. This high prevalence may confound MDD studies not accounting for history of childhood trauma. Further, in this work, effects of childhood trauma within MDD were considered on structure and metabolism, assessed simultaneously, in the same individual, allowing assessment of functional and structural differences.

Examining correlates of childhood trauma could aid in the adoption of preventative treatment. For example, cognitive behavioral therapy (CBT) has been associated with increases in volume in the hippocampus [62, 63] in PTSD. Therefore, CBT may be a useful in normalizing the structural and functional changes associated with mental health disorders [64, 65] or following childhood trauma. Similarly, psychotherapies [66, 67] and evidence-based treatment [68] led to observable functional and structural changes in depressed patients and a positive relationship between total cerebral volume and availability of ‘supportive listening’ in depressed adults was observed [69]. Potentially, treatment options targeting the neurobiological differences identified in this cohort could be used preventatively following trauma.

Although this study specifically focused on childhood trauma in the context of depression, the results may be applicable to other disorders as childhood trauma is a transdiagnostic risk factor, including for psychosis and schizophrenia [70, 71]. Similar to MDD, individuals with psychosis are more likely to have a history of childhood trauma than not [70]. Those with a history of trauma are also more likely to exhibit higher levels of depression or anxiety [71]. As such, future work will consider whether the neurobiological associations shown here exist across disorders or are specifically related to MDD. In such work, a non-psychiatric control group would be useful.

### Subcortical regions: amygdala & hippocampus (volume and metabolism)

The amygdala, implicated in emotional response, activates the HPA and autonomic nervous system when a stressor is present. A correlation between smaller amygdala volumes and an exaggerated glucocorticoid response to stress has been shown [20]. The hippocampus, a critical area which aids in memory and cognition [72], is regarded to play an important inhibitory role in terminating the HPA stress response [15, 21] and this region appears to be highly vulnerable to change following emotional distress [15, 18].

Numerous studies have consistently reported reduced amygdala and hippocampus volumes in those who experienced childhood trauma [15–19]. We similarly report a relationship between total CTQ score and volumes in these regions (Fig. 1H, J). Further, the robust finding in the amygdala survives Bonferroni correction. Based on this analysis, in childhood trauma within MDD, for each unit increase in childhood trauma severity amygdala volume is estimated to be lower by 7.44 mm^3^ (hippocampus volume is estimated to be lower by 9.57 mm^3^). While these values are <1% of the total volumes of these structures, over the range of CTQ scores reported here (26 to 100), this could result in an ~8% reduced hippocampal volume and ~17% reduced amygdala volume.

A similar effect was not observable when examining childhood trauma categorically (Supplementary Material, Table S1). This is likely because of the larger variance in the volumes of those without childhood trauma and potentially due to the challenge of distinguishing between low and moderate trauma levels. As seen in Fig. 1, in terms of total CTQ score, the low and moderate discrete childhood trauma levels reveal a fair amount of overlap.

Although childhood trauma effects on amygdala and hippocampus volume were not reported in the largest neuroimaging study in childhood trauma within MDD to date, the ENIGMA study [49], effects of medication on both these regions were reported (though amygdala effects did not survive multiple comparison correction). In contrast, all participants in this current study were medication free for at least 3 weeks prior to imaging. As medication may have variable effects on the brain [73–75], the most cautious analyses should be performed in the medication-free state as performed here. Moreover, in particular, the literature suggests that a 4-week medication washout period is a sufficient timeframe for reversal of structural changes associated with treatment [76].

Interestingly, examining childhood trauma categorically (Supplementary Material, Table S1) suggests amygdala metabolism may be higher in participants with severe childhood trauma compared to those with low/moderate trauma. These findings point to amygdala hyperactivity in those experiencing adversity. The magnitude of significant difference in amygdala metabolism between low and severe childhood trauma levels within MDD was 0.33 mg/(min*100 mL) (~11% of the average). However, as these results do not survive Bonferroni correction, they will need to be examined in a larger cohort. If validated, these differences could aid in identifying people at risk of MDD, as most metabolic changes occur prior to those of structural [77], therefore identifying trends like that seen in the amygdala may assist in the early detection and intervention.

### Cortical thickness versus volume

Both thickness and volume structural measures were considered for the two cortical regions in this analysis. Cortical thickness is the average thickness of the gray matter that lies between the white matter surface and the pial surface. Volume is a composite of thickness and surface area, but it is more closely related to surface area. However, cortical thickness and surface area measurements are independent both regionally and globally in the brain, and are genetically and phenotypically uncorrelated [50]. For example, histological parameters involved in age-related changes appear to be associated with cortical thickness to a greater extent than volume [78, 79].

Volume, however, is a more comprehensive measure that incorporates both thickness and cortical folding. Some factors that affect the cortex will affect both thickness and area, thus volume serves as a value to measure the effects of non-specific factors in the cortex [78]. Changes in the volume of white matter, gray matter, and cerebrospinal fluid are critical in identifying diseases and are repeatedly found to play a significant role in the monitoring and evaluation of treatments for various neurodegenerative diseases [80].

The differences in these measures are reflected in this study’s results. Only DLPFC thickness was correlated with DLPFC metabolism. This may suggest that cortical thickness is more closely coupled with function in this region, which may explain the lack of significant findings between volume of the cortical regions and childhood trauma on the discrete or continuous scales. However, it is important to keep in mind that DLPFC metabolism was not associated with continuous CTQ score and no differences in DLPFC metabolism were found across discrete trauma levels.

In addition to being a large region, the prefrontal cortex is heavily involved in executive function, attention, and memory. It has an expansive network and is connected to regions such at the hippocampus, dorsal caudate nucleus, lateral thalamus, and the neocortex [81]. Moreover, the DLPFC is one of the last cortical regions to mature functionally and structurally [82]. These properties may explain its unique structure / function relationship; however, more study into this coupling is needed. This should include examining this relationship in a control cohort.

### Cortical regions: anterior cingulate cortex and dorsolateral prefrontal cortex (cortical thickness, volume, and metabolism)

In the analysis of cortical regions, DLPFC thickness was correlated with continuous CTQ score (Fig. 1F) and differences were found between none and moderate childhood trauma levels (Table S1). These findings did not survive Bonferroni correction. In a larger sample size, these findings may have remained significant. However, the clinical significance would still need to be further examined. For example, the significant differences in DLPFC thickness were, on average, 0.08 mm (between no childhood trauma and moderate childhood trauma in MDD) and 0.0015 mm per point of CTQ. This accounts for <5% of the average thickness.

### Limitations

There are some limitations in our study. Our sample included only depressed adults within a limited age range. One advantage of examining a more homogenous sample, however, is the reduction of confounding variables. For example, socioeconomic status has been shown to affect neurobiology [83–86], specifically the hippocampus [87, 88]. While we do not correct for that effect here, the demographics of our participants were fairly uniform. For example, ~5% completed high school or its equivalent, ~64% attended college, ~18% completed college and ~13% completed post graduate training.

The CTQ is a retrospective measure, which can suffer from inaccuracies due to recall bias [89, 90], and does not control for the time within childhood the trauma occurred. Moreover, as this study did not examine participants longitudinally, only association, not causation can be examined. As such, these structural/functional differences could have existed prior to the childhood trauma. For example, there is debate as to whether stress reduces hippocampus volumes or if low hippocampus volumes predispose one to stress [91]. A smaller hippocampus may less efficiently influence the HPA axis and also negatively influence how individuals deal with new challenges and stressors [92]. This causation can only be determined through a longitudinal study examining participants prior to the introduction of such stressors.

Additionally, to prevent the reduction of statistical power due to multiple comparisons, analysis was restricted to four regions that have been implicated in the stress response as well as MDD/childhood trauma. Future studies can consider more regions such as the caudate nucleus implicated in the ENIGMA study.

Regarding the imaging outcome measures, due to the resolution of PET, it is possible that estimates of metabolism were affected by regional thickness, as a result of the partial volume effect. However, given the small range of thickness variation, as described above, this is unlikely to have affected study results.

Even though limitations did exist, several advantages should be noted to ensure the validity of the conclusions reached in this study. The current study involved a large cohort of participants (*N* > 80). All participants were medication free at least 3 weeks before imaging. Further, childhood trauma was examined both continuously and categorically and all analyses were computed by rigorous statistical analyses. Cortical thickness and volume was assessed automatically by Freesurfer and brain metabolism was calculated using a fully quantitative technique, involving blood sampling and dynamic PET imaging, instead of semi-quantitative methods [93–96] (e.g., normalized regional uptake), which are affected by many confounding factors for which there is no correction [93–95].

## Conclusions

It is critically important to examine the correlates of childhood trauma within MDD, because of the high prevalence of childhood trauma in those with MDD. Without understanding this relationship, MDD-control comparisons will be confounded by effects of childhood trauma. This, along with potential confounding factors of medication, may explain equivocal results on structural differences examined in MDD to date. By imaging participants with MDD who are medication free, and accounting for childhood trauma, this study was the first to quantify the relationship between increasing childhood trauma and volume in the amygdala, as well as the relationship between thickness and metabolism in the DLPFC. In the future, such multimodal approaches may be used to examine whether there are any interactions between structure and metabolism such as whether the relationship between structure and metabolism is moderated by childhood maltreatment. These findings may aid in the developing treatment targets for the prevention of MDD following childhood trauma.

## Supplementary information

Supplementary Material

Figure S1

## Data Availability

The data that support the findings of this study are available from the senior author, CD, upon request.

## References

[1] Fitzgerald PB, Laird AR, Maller J, Daskalakis ZJ (2008). A meta-analytic study of changes in brain activation in depression. Hum Brain Mapp.

[2] Kaplow JB, Widom CS (2007). Age of onset of child maltreatment predicts long-term mental health outcomes. J Abnorm Psychol.

[3] Martins CM, Von Werne Baes C, Tofoli SM, Juruena MF (2014). Emotional abuse in childhood is a differential factor for the development of depression in adults. J Nerv Ment Dis.

[4] Kessler RC, Magee WJ (1994). Childhood family violence and adult recurrent depression. J Health Soc Behav.

[5] Devi F, Shahwan S, Teh WL, Sambasivam R, Zhang YJ, Lau YW (2019). The prevalence of childhood trauma in psychiatric outpatients. Ann Gen Psychiatry.

[6] Negele A, Kaufhold J, Kallenbach L, Leuzinger-Bohleber M (2015). Childhood Trauma and its relation to chronic depression in adulthood. Depress Res Treat.

[7] Humphreys KL, LeMoult J, Wear JG, Piersiak HA, Lee A, Gotlib IH (2020). Child maltreatment and depression: a meta-analysis of studies using the Childhood Trauma Questionnaire. Child Abus Negl.

[8] Teicher MH, Samson JA, Anderson CM, Ohashi K (2016). The effects of childhood maltreatment on brain structure, function and connectivity. Nat Rev Neurosci.

[9] Parr LA, Boudreau M, Hecht E, Winslow JT, Nemeroff CB, Sanchez MM (2012). Early life stress affects cerebral glucose metabolism in adult rhesus monkeys (Macaca mulatta). Dev Cogn Neurosci.

[10] Harkness KL, Bruce AE, Lumley MN (2006). The role of childhood abuse and neglect in the sensitization to stressful life events in adolescent depression. J Abnorm Psychol.

[11] Fu CH, Williams SC, Cleare AJ, Brammer MJ, Walsh ND, Kim J (2004). Attenuation of the neural response to sad faces in major depression by antidepressant treatment: a prospective, event-related functional magnetic resonance imaging study. Arch Gen Psychiatry.

[12] Anand A, Li Y, Wang Y, Wu J, Gao S, Bukhari L (2005). Activity and connectivity of brain mood regulating circuit in depression: a functional magnetic resonance study. Biol Psychiatry.

[13] Adolphs R (2003). Cognitive neuroscience of human social behaviour. Nat Rev Neurosci.

[14] Hari R, Kujala MV (2009). Brain basis of human social interaction: from concepts to brain imaging. Physiol Rev.

[15] McEwen BS, Nasca C, Gray JD (2016). Stress effects on neuronal structure: hippocampus, amygdala, and prefrontal cortex. Neuropsychopharmacology.

[16] Vyas A, Mitra R, Shankaranarayana Rao BS, Chattarji S (2002). Chronic stress induces contrasting patterns of dendritic remodeling in hippocampal and amygdaloid neurons. J Neurosci.

[17] Carballedo A, Morris D, Zill P, Fahey C, Reinhold E, Meisenzahl E (2013). Brain-derived neurotrophic factor Val66Met polymorphism and early life adversity affect hippocampal volume. Am J Med Genet B Neuropsychiatr Genet.

[18] Kim EJ, Pellman B, Kim JJ (2015). Stress effects on the hippocampus: a critical review. Learn Mem.

[19] Hanson JL, Nacewicz BM, Sutterer MJ, Cayo AA, Schaefer SM, Rudolph KD (2015). Behavioral problems after early life stress: contributions of the hippocampus and amygdala. Biol Psychiatry.

[20] Yang RJ, Mozhui K, Karlsson RM, Cameron HA, Williams RW, Holmes A (2008). Variation in mouse basolateral amygdala volume is associated with differences in stress reactivity and fear learning. Neuropsychopharmacology.

[21] van Bodegom M, Homberg JR, Henckens M (2017). Modulation of the hypothalamic-pituitary-adrenal axis by early life stress exposure. Front Cell Neurosci.

[22] Demir-Lira OE, Voss JL, O’Neil JT, Briggs-Gowan MJ, Wakschlag LS, Booth JR (2016). Early-life stress exposure associated with altered prefrontal resting-state fMRI connectivity in young children. Dev Cogn Neurosci.

[23] Zhang K, Zhu Y, Zhu Y, Wu S, Liu H, Zhang W (2016). Molecular, functional, and structural imaging of major depressive disorder. Neurosci Bull.

[24] Jaworska N, Yucel K, Courtright A, MacMaster FP, Sembo M, MacQueen G (2016). Subgenual anterior cingulate cortex and hippocampal volumes in depressed youth: The role of comorbidity and age. J Affect Disord.

[25] van Tol MJ, van der Wee NJ, van den Heuvel OA, Nielen MM, Demenescu LR, Aleman A (2010). Regional brain volume in depression and anxiety disorders. Arch Gen Psychiatry.

[26] Grieve SM, Korgaonkar MS, Koslow SH, Gordon E, Williams LM (2013). Widespread reductions in gray matter volume in depression. Neuroimage Clin.

[27] Cohen RA, Grieve S, Hoth KF, Paul RH, Sweet L, Tate D (2006). Early life stress and morphometry of the adult anterior cingulate cortex and caudate nuclei. Biol Psychiatry.

[28] Staffaroni AM, Melrose RJ, Leskin LP, Riskin-Jones H, Harwood D, Mandelkern M (2017). The functional neuroanatomy of verbal memory in Alzheimer’s disease: [(18)F]-Fluoro-2-deoxy-D-glucose positron emission tomography (FDG-PET) correlates of recency and recognition memory. J Clin Exp Neuropsychol.

[29] Verger A, Roman S, Chaudat RM, Felician O, Ceccaldi M, Didic M (2017). Changes of metabolism and functional connectivity in late-onset deafness: Evidence from cerebral (18)F-FDG-PET. Hear Res.

[30] Taylor SE, Eisenberger NI, Saxbe D, Lehman BJ, Lieberman MD (2006). Neural responses to emotional stimuli are associated with childhood family stress. Biol Psychiatry.

[31] Wang L, Dai Z, Peng H, Tan L, Ding Y, He Z (2014). Overlapping and segregated resting-state functional connectivity in patients with major depressive disorder with and without childhood neglect. Hum Brain Mapp.

[32] van Harmelen AL, van Tol MJ, Dalgleish T, van der Wee NJ, Veltman DJ, Aleman A (2014). Hypoactive medial prefrontal cortex functioning in adults reporting childhood emotional maltreatment. Soc Cogn Affect Neurosci.

[33] Yamamoto T, Toki S, Siegle GJ, Takamura M, Takaishi Y, Yoshimura S (2017). Increased amygdala reactivity following early life stress: a potential resilience enhancer role. BMC Psychiatry.

[34] Davis M, Whalen PJ (2001). The amygdala: vigilance and emotion. Mol Psychiatry.

[35] Merz EC, He X, Noble KG, Pediatric Imaging, Neurocognition, and Genetics Study (2018). Anxiety, depression, impulsivity, and brain structure in children and adolescents. Neuroimage Clin.

[36] Belleau EL, Treadway MT, Pizzagalli DA (2019). The impact of stress and major depressive disorder on hippocampal and medial prefrontal cortex morphology. Biol Psychiatry.

[37] Phillips JL, Batten LA, Tremblay P, Aldosary F, Blier PA. Prospective, longitudinal study of the effect of remission on cortical thickness and hippocampal volume in patients with treatment-resistant depression. Int J Neuropsychopharmacol. 2015;18:pyv037. 10.1093/ijnp/pyv037 PMC4571636 25829180

[38] Zuo Z, Ran S, Wang Y, Li C, Han Q, Tang Q (2018). Altered structural covariance among the dorsolateral prefrontal cortex and amygdala in treatment-naive patients with major depressive disorder. Front Psychiatry.

[39] Malykhin NV, Carter R, Hegadoren KM, Seres P, Coupland NJ (2012). Fronto-limbic volumetric changes in major depressive disorder. J Affect Disord.

[40] Colloby SJ, Firbank MJ, Vasudev A, Parry SW, Thomas AJ, O’Brien JT (2011). Cortical thickness and VBM-DARTEL in late-life depression. J Affect Disord.

[41] Perlman G, Bartlett E, DeLorenzo C, Weissman M, McGrath P, Ogden T (2017). Cortical thickness is not associated with current depression in a clinical treatment study. Hum Brain Mapp.

[42] Yang J, Zhang M, Ahn H, Zhang Q, Jin TB, Li I (2018). Development and evaluation of a multimodal marker of major depressive disorder. Hum Brain Mapp.

[43] Winter NR, Leenings R, Ernsting J, Sarink K, Fisch L, Emden D, et al. Quantifying deviations of brain structure and function in major depressive disorder across neuroimaging modalities. JAMA Psychiatry. 2022;79:879–88. 10.1001/jamapsychiatry.2022.1780 PMC9330277 35895072

[44] Baeken C, Wu GR, De Raedt R (2018). Dorsomedial frontal cortical metabolic differences of comorbid generalized anxiety disorder in refractory major depression: A [(18)F] FDG PET brain imaging study. J Affect Disord.

[45] Su L, Cai Y, Xu Y, Dutt A, Shi S, Bramon E (2014). Cerebral metabolism in major depressive disorder: a voxel-based meta-analysis of positron emission tomography studies. BMC Psychiatry.

[46] Kennedy SH, Evans KR, Kruger S, Mayberg HS, Meyer JH, McCann S (2001). Changes in regional brain glucose metabolism measured with positron emission tomography after paroxetine treatment of major depression. Am J Psychiatry.

[47] Chaney A, Carballedo A, Amico F, Fagan A, Skokauskas N, Meaney J (2014). Effect of childhood maltreatment on brain structure in adult patients with major depressive disorder and healthy participants. J Psychiatry Neurosci.

[48] Saleh A, Potter GG, McQuoid DR, Boyd B, Turner R, MacFall JR (2017). Effects of early life stress on depression, cognitive performance and brain morphology. Psychol Med.

[49] Frodl T, Janowitz D, Schmaal L, Tozzi L, Dobrowolny H, Stein DJ (2017). Childhood adversity impacts on brain subcortical structures relevant to depression. J Psychiatr Res.

[50] Winkler AM, Kochunov P, Blangero J, Almasy L, Zilles K, Fox PT (2010). Cortical thickness or grey matter volume? The importance of selecting the phenotype for imaging genetics studies. Neuroimage.

[51] Tozzi L, Garczarek L, Janowitz D, Stein DJ, Wittfeld K, Dobrowolny H (2020). Interactive impact of childhood maltreatment, depression, and age on cortical brain structure: mega-analytic findings from a large multi-site cohort. Psychol Med.

[52] Hill KR, Gardus JD, Bartlett EA, Perlman G, Parsey RV, DeLorenzo C (2021). Measuring brain glucose metabolism in order to predict response to antidepressant or placebo: A randomized clinical trial. Neuroimage Clin.

[53] Narayan GA, Hill KR, Wengler K, He X, Wang J, Yang J, et al. Does the change in glutamate to GABA ratio correlate with change in depression severity? A randomized, double-blind clinical trial. Mol Psychiatry. 2022. 10.1038/s41380-022-01730-4. 10.1038/s41380-022-01730-4 PMC9712215 35982258

[54] Montgomery SA, Asberg M (1979). A new depression scale designed to be sensitive to change. Br J Psychiatry.

[55] Bernstein DP, Fink L, Handelsman L, Foote J, Lovejoy M, Wenzel K (1994). Initial reliability and validity of a new retrospective measure of child abuse and neglect. Am J Psychiatry.

[56] Frodl T, Reinhold E, Koutsouleris N, Reiser M, Meisenzahl EM (2010). Interaction of childhood stress with hippocampus and prefrontal cortex volume reduction in major depression. J Psychiatr Res.

[57] Iscan Z, Jin TB, Kendrick A, Szeglin B, Lu H, Trivedi M (2015). Test-retest reliability of freesurfer measurements within and between sites: Effects of visual approval process. Hum brain Mapp.

[58] Desikan RS, Segonne F, Fischl B, Quinn BT, Dickerson BC, Blacker D (2006). An automated labeling system for subdividing the human cerebral cortex on MRI scans into gyral based regions of interest. NeuroImage.

[59] Izquierdo-Garcia D, Hansen AE, Forster S, Benoit D, Schachoff S, Furst S (2014). An SPM8-based approach for attenuation correction combining segmentation and nonrigid template formation: application to simultaneous PET/MR brain imaging. J Nucl Med.

[60] Wang Y, Xu Q, Luo J, Hu M, Zuo C (2019). Effects of age and sex on subcortical volumes. Front Aging Neurosci.

[61] Bartlett EA, DeLorenzo C, Sharma P, Yang J, Zhang M, Petkova E (2018). Pretreatment and early-treatment cortical thickness is associated with SSRI treatment response in major depressive disorder. Neuropsychopharmacology.

[62] Levy-Gigi E, Szabo C, Kelemen O, Keri S (2013). Association among clinical response, hippocampal volume, and FKBP5 gene expression in individuals with posttraumatic stress disorder receiving cognitive behavioral therapy. Biol Psychiatry.

[63] Rubin M, Shvil E, Papini S, Chhetry BT, Helpman L, Markowitz JC (2016). Greater hippocampal volume is associated with PTSD treatment response. Psychiatry Res Neuroimaging.

[64] Mansson KN, Salami A, Frick A, Carlbring P, Andersson G, Furmark T (2016). Neuroplasticity in response to cognitive behavior therapy for social anxiety disorder. Transl Psychiatry.

[65] Chattopadhyay S, Tait R, Simas T, van Nieuwenhuizen A, Hagan CC, Holt RJ (2017). Cognitive behavioral therapy lowers elevated functional connectivity in depressed adolescents. EBioMedicine.

[66] Weingarten CP, Strauman TJ (2015). Neuroimaging for psychotherapy research: current trends. Psychother Res.

[67] Klimes-Dougan B, Basgoze Z, Mueller B, Wiglesworth A, Carosella KA, Westlund Schreiner M, et al. Structural and functional neural correlates of treatment response for interpersonal psychotherapy for depressed adolescents. J Clin Med. 2022;11:1878. 10.3390/jcm11071878 PMC8999886 35407493

[68] Vanderzee KL, Sigel BA, Pemberton JR, John SG (2019). Treatments for early childhood trauma: decision considerations for clinicians. J Child Adolesc Trauma.

[69] Salinas J, O’Donnell A, Kojis DJ, Pase MP, DeCarli C, Rentz DM (2021). Association of social support with brain volume and cognition. JAMA Netw Open.

[70] Gaudiano BA, Zimmerman M (2010). The relationship between childhood trauma history and the psychotic subtype of major depression. Acta Psychiatr Scand.

[71] Schafer I, Fisher HL (2011). Childhood trauma and psychosis-what is the evidence?. Dialogues Clin Neurosci.

[72] Lisman J, Buzsaki G, Eichenbaum H, Nadel L, Ranganath C, Redish AD (2017). Viewpoints: how the hippocampus contributes to memory, navigation and cognition. Nat Neurosci.

[73] Nemati S, Abdallah CG. Increased cortical thickness in patients with major depressive disorder following antidepressant treatment. Chronic Stress (Thousand Oaks). 2020;4:2470547019899962. 10.1177/2470547019899962 PMC6959134 31938760

[74] Dusi N, Barlati S, Vita A, Brambilla P (2015). Brain structural effects of antidepressant treatment in major depression. Curr Neuropharmacol.

[75] An J, Wang L, Li K, Zeng Y, Su Y, Jin Z (2017). Differential effects of antidepressant treatment on long-range and short-range functional connectivity strength in patients with major depressive disorder. Sci Rep.

[76] Hoffman DA, Schiller M, Greenblatt JM, Iosifescu DV (2011). Polypharmacy or medication washout: an old tool revisited. Neuropsychiatr Dis Treat.

[77] Camandola S, Mattson MP (2017). Brain metabolism in health, aging, and neurodegeneration. EMBO J.

[78] Lemaitre H, Goldman AL, Sambataro F, Verchinski BA, Meyer-Lindenberg A, Weinberger DR (2012). Normal age-related brain morphometric changes: nonuniformity across cortical thickness, surface area and gray matter volume?. Neurobiol Aging.

[79] Fjell AM, Westlye LT, Amlien I, Espeseth T, Reinvang I, Raz N (2009). High consistency of regional cortical thinning in aging across multiple samples. Cereb Cortex.

[80] Zeinali R, Keshtkar A, Zamani A, Gharehaghaji N (2017). Brain volume estimation enhancement by morphological image processing tools. J Biomed Phys Eng.

[81] Siddiqui SV, Chatterjee U, Kumar D, Siddiqui A, Goyal N (2008). Neuropsychology of prefrontal cortex. Indian J Psychiatry.

[82] Gogtay N, Giedd JN, Lusk L, Hayashi KM, Greenstein D, Vaituzis AC (2004). Dynamic mapping of human cortical development during childhood through early adulthood. Proc Natl Acad Sci USA.

[83] Dufford AJ, Evans GW, Liberzon I, Swain JE, Kim P (2021). Childhood socioeconomic status is prospectively associated with surface morphometry in adulthood. Dev Psychobiol.

[84] Javanbakht A, Kim P, Swain JE, Evans GW, Phan KL, Liberzon I. Sex-specific effects of childhood poverty on neurocircuitry of processing of emotional cues: a neuroimaging study. Behav Sci (Basel). 2016;6:28. 10.3390/bs6040028 PMC5197941 27973443

[85] Javanbakht A, King AP, Evans GW, Swain JE, Angstadt M, Phan KL (2015). Childhood poverty predicts adult amygdala and frontal activity and connectivity in response to emotional faces. Front Behav Neurosci.

[86] Sripada RK, Swain JE, Evans GW, Welsh RC, Liberzon I (2014). Childhood poverty and stress reactivity are associated with aberrant functional connectivity in default mode network. Neuropsychopharmacology.

[87] Duval ER, Garfinkel SN, Swain JE, Evans GW, Blackburn EK, Angstadt M (2017). Childhood poverty is associated with altered hippocampal function and visuospatial memory in adulthood. Dev Cogn Neurosci.

[88] Liberzon I, Ma ST, Okada G, Ho SS, Swain JE, Evans GW (2015). Childhood poverty and recruitment of adult emotion regulatory neurocircuitry. Soc Cogn Affect Neurosci.

[89] Hardt J, Rutter M (2004). Validity of adult retrospective reports of adverse childhood experiences: review of the evidence. J Child Psychol Psychiatry.

[90] Coughlin SS (1990). Recall bias in epidemiologic studies. J Clin Epidemiol.

[91] Lindgren L, Bergdahl J, Nyberg L (2016). Longitudinal evidence for smaller hippocampus volume as a vulnerability factor for perceived stress. Cereb Cortex.

[92] McEwen BS, Gianaros PJ (2011). Stress- and allostasis-induced brain plasticity. Annu Rev Med.

[93] Keyes JW (1995). SUV: standard uptake or silly useless value?. J Nucl Med.

[94] Thie JA (2004). Understanding the standardized uptake value, its methods, and implications for usage. J Nucl Med.

[95] Boellaard R, Krak NC, Hoekstra OS, Lammertsma AA (2004). Effects of noise, image resolution, and ROI definition on the accuracy of standard uptake values: a simulation study. J Nucl Med.

[96] Cheebsumon P, Velasquez LM, Hoekstra CJ, Hayes W, Kloet RW, Hoetjes NJ (2011). Measuring response to therapy using FDG PET: semi-quantitative and full kinetic analysis. Eur J Nucl Med Mol Imaging.

