# Supplementary material for: Evaluation of brain structure and metabolism in currently depressed adults with a history of childhood trauma
Source: Transl Psychiatry. 2022 Sep 17;12:392. doi: 10.1038/s41398-022-02153-z (PMC9482635; doi:10.1038/s41398-022-02153-z)
Supplement: Supplementary file 1 — Supplementary Material [file 41398_2022_2153_MOESM1_ESM.docx]

Supplementary Material

To assess correlates of childhood trauma within MDD on brain biology, categorical analysis in which childhood trauma levels were divided into four levels as well as using the CTQ score as a continuous measure were considered. The former provides additional resolution on the effects of childhood trauma rather than a simple division into two groups (childhood trauma present / not present) and can uncover relationships with biology complimentary to the analysis using the CTQ score as a continuous measure. For example, as can be seen by the colors in Figure 1, childhood trauma categorized as severe (red) does not always correspond to the highest total CTQ score. Therefore, the effects of experiencing severe childhood trauma in one category may be lost by considering total CTQ score only. For this reason, using both analyses provide a more complete picture of the potential effects of childhood trauma on critical regions involved in the response to stress - the DLPFC, ACC, hippocampus, and amygdala.

*Analysis using childhood trauma severity as a categorical variable*

Of the variables shown in Figure S1, DLPFC thickness (Figure S1F) showed significant differences between none and moderate childhood trauma levels and amygdala metabolism (Figure S1B) showed significant differences between low/moderate and severe trauma. None of these comparisons remained significant following Bonferroni correction (Table S1). Including depression severity as a covariate did not change model results.

**Figure S1**

Figure S1. Boxplots for Regional Volume/Thickness/Metabolism by Level of Childhood Trauma Severity. (*) = p < 0.05 uncorrected. ACC, Anterior Cingulate Cortex; DLPFC, Dorsolateral Prefrontal Cortex; HIP, Hippocampus; AMY, Amygdala.

Table S1. Estimated coefficient or difference with the 95% Confidence Interval (CI) of explanatory variables based on linear mixed models after controlling for sex, age, and age^2^. The discrete childhood trauma levels are defined as follows: none (0): participants with ‘none’ (0) across all trauma categories; low (1): participants who have at least one ‘low’ value in a trauma category and no ‘moderate’ or ‘severe’ scores; moderate (2): participants who have at least one ‘moderate’ value in a trauma category and no severe scores; severe (3): participants who have at least one ‘severe’ value in a trauma category. (See Table 1) ACC, Anterior Cingulate Cortex; DLPFC, Dorsolateral Prefrontal Cortex; HIP, Hippocampus; AMY, Amygdala.

| **Outcome** | **Region** | **Childhood Trauma Level** | **Estimated coefficient or difference and 95% CI** | **Pairwise p-value^a^** | **p-value^b^** |
| --- | --- | --- | --- | --- | --- |
| Thickness^c^  (*n*=81) | ACC | 0 vs 1 | 0.03 (-0.09, 0.15) | 0.6483 | 0.7385 |
|  |  | 0 vs 2 | 0.07 (-0.03, 0.17) | 0.1641 |  |
|  |  | 0 vs 3 | 0.03 (-0.06, 0.13) | 0.4925 |  |
|  |  | 1 vs 2 | 0.04 (-0.07, 0.15) | 0.4343 |  |
|  |  | 1 vs 3 | 0.01 (-0.10, 0.11) | 0.9171 |  |
|  |  | 2 vs 3 | -0.04 (-0.12, 0.04) | 0.3473 |  |
|  | DLPFC | 0 vs 1 | 0.01 (-0.07, 0.10) | 0.7371 |  |
|  |  | 0 vs 2 | 0.08 (0.01, 0.15) | 0.0253* |  |
|  |  | 0 vs 3 | 0.0664 (-0.0002, 0.1330) | 0.0506 |  |
|  |  | 1 vs 2 | 0.06 (-0.01, 0.14) | 0.0954 |  |
|  |  | 1 vs 3 | 0.05 (-0.02, 0.12) | 0.1546 |  |
|  |  | 2 vs 3 | -0.01 (-0.07, 0.04) | 0.6405 |  |
| Volume^c^  (*n*=80) | ACC | 0 vs 1 | -0.54 (-1.37, 0.29) | 0.2022 | 0.7847 |
|  |  | 0 vs 2 | -0.14 (-0.82, 0.55) | 0.6936 |  |
|  |  | 0 vs 3 | 0.02 (-0.64, 0.68) | 0.9606 |  |
|  |  | 1 vs 2 | 0.40 (-0.34, 1.13) | 0.2831 |  |
|  |  | 1 vs 3 | 0.55 (-0.15, 1.26) | 0.1232 |  |
|  |  | 2 vs 3 | 0.15 (-0.38, 0.69) | 0.5723 |  |
|  | AMY | 0 vs 1 | -0.04 (-0.52, 0.43) | 0.8568 |  |
|  |  | 0 vs 2 | 0.16 (-0.23, 0.55) | 0.4118 |  |
|  |  | 0 vs 3 | 0.29 (-0.08, 0.66) | 0.1188 |  |
|  |  | 1 vs 2 | 0.20 (-0.22, 0.63) | 0.3415 |  |
|  |  | 1 vs 3 | 0.34 (-0.06, 0.74) | 0.0970 |  |
|  |  | 2 vs 3 | 0.13 (-0.17, 0.43) | 0.3812 |  |
|  | DLPFC | 0 vs 1 | -0.16 (-1.56, 1.24) | 0.8167 |  |
|  |  | 0 vs 2 | 0.25 (-0.91, 1.42) | 0.6688 |  |
|  |  | 0 vs 3 | 0.14 (-0.98, 1.26) | 0.8067 |  |
|  |  | 1 vs 2 | 0.41 (-0.82, 1.65) | 0.5067 |  |
|  |  | 1 vs 3 | 0.30 (-0.89, 1.49) | 0.6157 |  |
|  |  | 2 vs 3 | -0.11 (-1.02, 0.80) | 0.8045 |  |
|  | HIP | 0 vs 1 | -0.12 (-0.65, 0.41) | 0.6616 |  |
|  |  | 0 vs 2 | -0.10 (-0.53, 0.34) | 0.6604 |  |
|  |  | 0 vs 3 | 0.09 (-0.32, 0.51) | 0.6592 |  |
|  |  | 1 vs 2 | 0.02 (-0.45, 0.49) | 0.9312 |  |
|  |  | 1 vs 3 | 0.21 (-0.24, 0.66) | 0.3545 |  |
|  |  | 2 vs 3 | 0.19 (-0.15, 0.53) | 0.2713 |  |
| Metabolism  (*n*=70) | ACC | 0 vs 1 | 0.49 (-0.22, 1.20) | 0.1701 | 0.3256 |
|  |  | 0 vs 2 | 0.33 (-0.29, 0.95) | 0.2860 |  |
|  |  | 0 vs 3 | 0.03 (-0.55, 0.62) | 0.9055 |  |
|  |  | 1 vs 2 | -0.16 (-0.80, 0.48) | 0.6181 |  |
|  |  | 1 vs 3 | -0.46 (-1.06, 0.15) | 0.1358 |  |
|  |  | 2 vs 3 | -0.30 (-0.80, 0.20) | 0.2381 |  |
|  | AMY | 0 vs 1 | 0.25 (-0.14, 0.64) | 0.2037 |  |
|  |  | 0 vs 2 | 0.21 (-0.13, 0.55) | 0.2167 |  |
|  |  | 0 vs 3 | -0.08 (-0.40, 0.24) | 0.6085 |  |
|  |  | 1 vs 2 | -0.04 (-0.40, 0.31) | 0.8153 |  |
|  |  | 1 vs 3 | -0.3335 (-0.6640, -0.0030) | 0.0480* |  |
|  |  | 2 vs 3 | -0.29 (-0.56, -0.02) | 0.0359* |  |
|  | DLPFC | 0 vs 1 | 0.56 (-0.26, 1.38) | 0.1756 |  |
|  |  | 0 vs 2 | 0.55 (-0.16, 1.27) | 0.1260 |  |
|  |  | 0 vs 3 | 0.05 (-0.62, 0.73) | 0.8763 |  |
|  |  | 1 vs 2 | -0.01 (-0.75, 0.73) | 0.9833 |  |
|  |  | 1 vs 3 | -0.51 (-1.21, 0.19) | 0.1514 |  |
|  |  | 2 vs 3 | -0.50 (-1.08, 0.08) | 0.0877 |  |
|  | HIP | 0 vs 1 | 0.23 (-0.21, 0.68) | 0.2936 |  |
|  |  | 0 vs 2 | 0.26 (-0.12, 0.64) | 0.1762 |  |
|  |  | 0 vs 3 | -0.03 (-0.39, 0.33) | 0.8596 |  |
|  |  | 1 vs 2 | 0.03 (-0.37, 0.43) | 0.8941 |  |
|  |  | 1 vs 3 | -0.27 (-0.64, 0.11) | 0.1603 |  |
|  |  | 2 vs 3 | -0.29 (-0.60, 0.02) | 0.0622 |  |
| * significant (p<=0.05) prior to Bonferroni correction  ^a^ p-value was from type 3 T test based on linear mixed models.  ^b^ p-value was from type 3 F test based on linear mixed models.  ^c^ Cube root transformation was used for volume to meet the normality assumption of the linear mixed model.  Note: Unstructured variance-covariance structure was selected for linear mixed model based on Akaike Information Criteria (AIC). | | | | | |
