# Supplementary figures and images for: Evaluation of brain structure and metabolism in currently depressed adults with a history of childhood trauma
Source: Transl Psychiatry. 2022 Sep 17;12:392. doi: 10.1038/s41398-022-02153-z (PMC9482635; doi:10.1038/s41398-022-02153-z)

# Childhood Trauma Severity Level

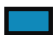

None

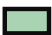

Low

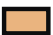

Moderate

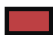

Severe

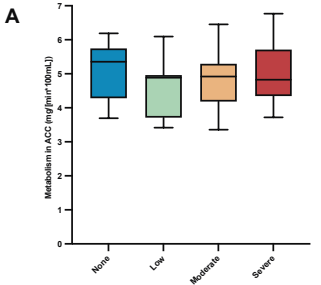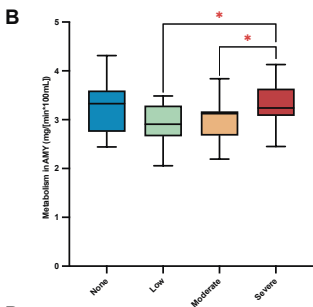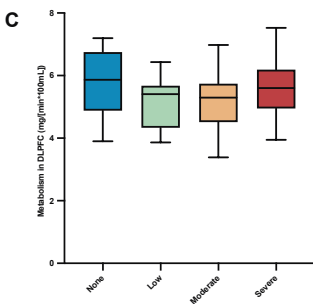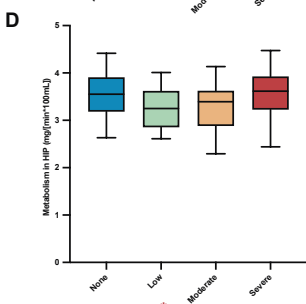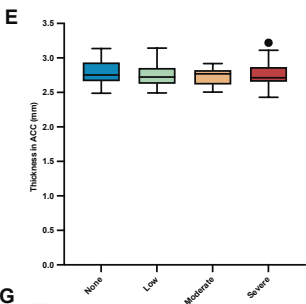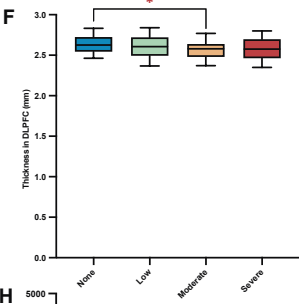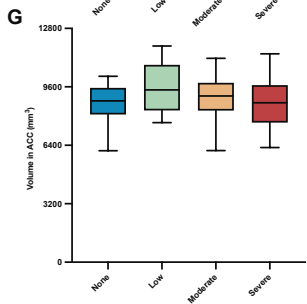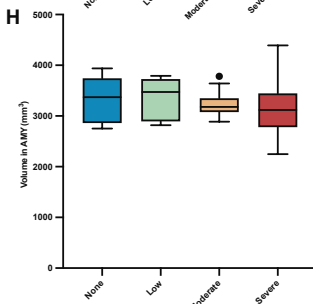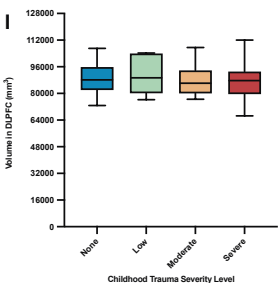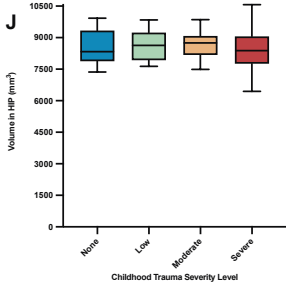

Supplement: Supplementary file 2 — Figure S1 [file 41398_2022_2153_MOESM2_ESM.pdf]
